# “They say that I have lost my integrity by breaking my virginity”: experiences of teen school going mothers in two schools in Lusaka Zambia

**DOI:** 10.1186/s12889-019-6394-0

**Published:** 2019-01-14

**Authors:** Shanny Nkwemu, Choolwe N. Jacobs, Oliver Mweemba, Anjali Sharma, Joseph M. Zulu

**Affiliations:** 1 Department of Health Promotion and Education, School of Public Health, University of Zambia, Lusaka, Zambia; 2 Department of Epidemiology and Biostatistics, School of Public Health, University of Zambia, Lusaka, Zambia; 3 Center for Infectious Disease Research in Zambia, Lusaka, Zambia; 4 Department of Public Health, School of Medicine, University of Zambia, P.O. Box 50110, Lusaka, Zambia

**Keywords:** Adolescents, Psychosocial, Experiences, Young mothers, Teenage, Coping strategies

## Abstract

**Background:**

Adolescent school-going mothers return to school in the rekindled hope of obtaining an education. However, their re-introduction into the school environment requires adequate support from teachers, fellow pupils, and the community. The purpose of this study was to explore the experiences of school-going mothers in Lusaka to understand their coping mechanism in the process of re-integration.

**Method:**

This is a qualitative case study. We conducted in-depth interviews with 24 school going mothers between the ages of 16–19, purposively selected from 2 schools in Lusaka district. Audio-recorded interviews were transcribed, coded using Nvivo 10 software and analysed using thematic analysis.

**Results:**

The girls reported experiencing stigmatization, discrimination, mockery and abuse from their teachers. Some community members labelled, humiliated, gossiped about and isolated the girls from their friends and classmates because of fear of ‘contamination’. Families forced some girls into early marriages making them feel rejected. These experiences resulted in low self-esteem, inferiority complex, poor performance in their academic work and identity crises in the young mothers. Therefore, because of the experiences the girls faced, they developed certain behaviours such as beer drinking, truancy and running away from home. They found it difficult to adjust to motherhood while doing their schoolwork.

**Conclusion:**

There is a need for the teachers to undergo training on how to handle young mothers and have a flexible time-table to accommodate adolescent school going mothers when they miss lessons to attend to their babies. Non-parenting school girls should be counseled so that they do not stigmatize adolescent mothers. Parents need to be educated on how to deal with adolescent mothers in the community. The guidance office should have a qualified psychosocial counselor to help create a conducive learning environment for adolescent mothers, by helping them with missed lessons and seeing to it that they are not stigmatized.

## Background

Globally, 101 million girls drop out of school annually because they get pregnant and 88% of these live in Africa and Asia [1], with only an estimated 34% returning to school after giving birth [2]. Adolescent motherhood is especially disruptive to the educational process of the girls with many teenage mothers leaving school, never to return. Those that return experience a disruption in the learning process both psychosocially and through lack of support from their social environment [3, 4]. Sub-Saharan Africa has the highest numbers of teenage mothers dropping out of school [5]. For instance, out of 13,634 million school girls who got pregnant in 2009, only 5517 million returned to school [6]. Girls dropping out of school threaten the national economics and labour market as well as the social and political development [7] of a country. Much research shows that the single predictor of family health is the completion of primary (elementary) school by girls normally from the ages of seven to 12 years.

The Forum for African Women Educationalist through their respective ministries of education have strategically lobbed to improve the education of the girls by allowing them to return to school after giving birth. However, implementation has not been straightforward because of the moral stigma associated with teenage motherhood [8]. Studies have shown that teen mothers experience unbearable pressure at school [9–11] such as shaming, self-stigma and being labelled as deviant [12]. Some teachers and administrators ignore the provision of equal opportunities for adolescent mothers by stigmatizing the girls in front of other learners [13] and did not give the girls the same chance to learn as they gave other learners. Instead, teachers utter hurtful words to discourage them from attending classes for fear that allowing teen mothers in school would appear to encourage immorality in school and wanted to protect the reputation of the school [14].

Adolescent mothers suffer from serious emotional disturbances with unfortunate social and health consequences. Teen mothers are prone to dropping out of school because their emotional feelings remain unattended to [15]. They are in danger of mood disorder which can lead to depression [16], or substance use and possibly sex work to support the habit. Consequently, they become more exposed to illicit behaviors. Experiences that adolescent school going mother face when they go back to school after giving birth in Zambia remains under-studied. Thus, the researcher sought to explore the experiences adolescent girls face when they go back to school after giving birth.

## Methods

### Study design

This was a qualitative case study conducted in Lusaka Zambia between October and November in 2015. Twenty-four adolescent school going mothers aged between 16 and 19 years were purposively selected from two secondary schools to achieve a better understanding of adolescent school going mothers’ experiences. The two schools were purposively selected with the help of the District Education Board Secretary office which provided information on schools with the highest numbers of adolescent school going mothers in the zone.

### Data collection

The 24 In-depth Interviews (IDIs) allowed face to face interaction between the researcher and adolescent school going mothers and the ability to clarify ambiguous questions or to seek more elaborations of incomplete answers. We chose to conduct IDIs over other possible methods because of the sensitivity of the topic and to protect the girls’ privacy. The use of an interview guide helped the researcher to explore and collect contextual information and young mothers’ ideas, thoughts and memories in their own words. A voice recorder was used with the participants to ensure that data collected was accurate and credible. During the interviews, field notes were taken to avoid reliance on recall which could have led to omission of important information in case of unseen events where the participant may nod or whisper.

### Data analysis

All interviews were conducted in English hence, there was no translation needed. Recorded files were downloaded on the computer to prepare for transcribing. Data were transcribed verbatim from the audio tape. Transcriptions were read for better comprehension where ideas about possible categories were written down as these came to mind. Similar topics were clustered together. Codes were written next to the appropriate segment text. Related topics were grouped together to reduce the number of categories. Transcripts were read and re-read the verbatim transcripts until specific codes emerged. These codes allowed the researcher to develop themes and categories. The codebook was developed and imported into Nvivo 10. All transcripts were imported into Nvivo 10 for analysis. Data was coded by themes (see Tables 1 and 2) with accompanying memos and participant quotes. Memos of key statements, ideas and attitudes were noted using as much as possible the words in the text.

**Table 1:** Demographic Characteristics of Adolescent School Going Mothers. As Table 1 below, 18 (75%) of the girls were 17–18 years old, about half were in grade 9 and 2 were married

Age	No. Per Age	Grade	No. Per Grade	Married
16 years	03	8	03	
17 years	10	9	11	
18 years	08	10	05	
19 years	03	11	03	
		12	02	02

**Table 2:** Major and Sub-themes of the Girls’ Experiences that Emerged from the Data

Major themes	Sub- themes
School- related challenges	Experience with teachers • Disapproving remarks by teachers • Teachers reminding the girls about their past • Lack of support from teachers • Mockery from the teachers Experience with pupils • Stigmatization • Isolation and rejection • Gossip from fellow pupils
Community-related challenges	• Multiple roles • Relationship with the community • Community fearing that girls will teach others immoral issues • Social insecurity
Family related challenges	• Support from home • Parenting and schooling • Forced early marriages • Labelling as deviances
Coping of young mothers	Behavioral Change • Skipping school days • Substance abuse • Staying away from home • Resilience • Spiritual Intervention

## Ethics approval

Ethical clearance (reference number 2015-June-026) was granted by Excellence in Research Ethics and Science (IRB No. 00005948), located along Addis Ababa road in Roads Park Lusaka-Zambia. Written permission was sought from District Education Board Secretary Offices (DEBS) to carry out the study in the mentioned schools. Written and verbal permission was obtained from the head-teachers of the two schools.

## Results

The primary source of information in this study were adolescent school going mothers aged 16 to 19. The girls were from grade eight to twelve and most of the girls were single except for two who were married. Main findings show that most of the girls experienced humiliation at school by teachers, classmates and peers. Some people in the community had a negative perception of these girls and were perceived as bad influences and others did not receive family support to cope with motherhood and public humiliation. For these reasons, some young mothers became resilient by embracing the ‘label’ of immoral (drinking) and taking intermittent breaks from school.

### Attitude of teachers toward teen mothers

While few adolescent school going mothers reported having been helped by some teachers on one or two occasions, a number of adolescent school going mothers during the interviews mentioned that teachers refused to assist adolescent mothers with the extra lessons when they missed class. Adolescent school going mothers reported that sometimes teachers would refer them to their fellow pupils who would not explain as much as the teacher

*“Teachers have never assisted me with extra lessons every time I miss the lessons to attend to my child when she is sick. They would tell me to ask my classmates to assist me with the missed lessons, but I used to feel shy to ask my friends… so that’s how I just stopped and forgot about it.*” (18 years grade 12 pupil).

However, a few participants acknowledged having been helped by their mathematics teacher on two occasions. Adolescent mothers mentioned that it was difficult to ask teachers for extra lessons as most of the teachers would demand payment towards the extra lesson.

*“Teachers would want to be paid for teaching extra lessons even if you had a genuine reason for not coming to school…I saw it from my Mathematics teacher the very first time I came back from maternity leave. I wanted some extra lessons on “Time calculation” then he told me that it was K100 per session. That is how I just stopped because I had no money to pay him.”*(19 years, grade 12 pupils).

### Mockery

When all the participants were asked on mockery, most adolescent school going mothers agreed to have mocked by some teachers at school. Adolescent school going mothers explained the situation to be so hurtful to the point that some young mothers contemplated going to neighboring schools where they were not known. Most participants perceived their teachers as being judgmental such that even seemingly good advice such as Voluntary Counselling and Testing (VCT) was seen as intended to emotionally hurt the learners.

*…“Some teachers would mention sensitive issues such as going for VCT, they may be saying it in good faith as an advice; but what hurt me most, was the way they would openly say it in class, though he didn’t mention my name, but my friends would laugh while looking at me…because one day he passed a bad comment when he saw me wearing a T-shirt written [Fridays uniform] “Virgin Power Virgin Pride”.”* (18 years grade 12 pupil).

### Lack of shame

Another recurring theme in respondent’s explanation for their experiences in school as adolescent mothers was that some teachers looked at adolescent mothers to have had lack of shame. Adolescent school going mothers during the discussion mentioned that teachers remained convinced that allowing adolescent mothers in school was undermining public moral standards of the education system.

*“Some teachers liked saying that I was stubborn because I came back to school after giving birth without feeling shy and pretending that all was well. They told me that I am a disgrace because I was degrading the standards of the school*.” (19 years grade 12 pupil).

### Stigmatization by fellow pupils

Furthermore, adolescent school going mothers mentioned how their fellow classmates stigmatized them. Adolescent school going mothers mentioned that some girls stated that schools were encouraging immoral behavior. The participants reported having been laughed at by their friends for having lost their virginity. The girls confirmed feeling neglected and judged by their friends.

*“Some of the girls in class would tease me…saying that the school was encouraging immorality by allowing mothers to continue with school when they are supposed to be home looking after their babies. What hurt me most was that the ones who liked saying that were my best friends whom I used to play with before I got pregnant. But now they would tell me that I have lost my integrity by breaking my virginity. My former best friend was the one in the forefront of saying that I have lost my virginity.”* (17 years old, grade 9 pupil).

Most participants highlighted that even if they were allowed in school as young mothers the treatment they got from both teachers and pupils was not good.

Almost all the girls further reported that some learners in school were always gossiping about their status and saying unpleasant things about them. Participants revealed that some girls would share young mothers’ private issues with other girls in the class. Adolescent school going mothers narrated how they felt when classmates were gossiping about the description of the fathers of their babies.

*“Some learners talk carelessly and even make up stories about me and my baby. One girl was telling others in class about the father of my baby, that he is a sugar daddy and that my child is ugly. The moment they saw me, they laughed and changed the story. I felt bad and wanted to beat her up. Then I thought of the punishment I was going to get for fighting in school.”* (16 years grade 8 pupil).

### Community fearing that young mothers will teach other girls bad morals

Some adolescents reported that some community members were not pleased to see adolescent mothers going back to school after giving birth. Adolescent mothers reported that the community’s argument was that adolescent mothers would teach their children immoral activities.

*“My aunty chased me when she discovered that I was pregnant…the people in the same compound used to say a lot of bad things about me because I continued going to school before I gave birth. Some community members would even pass bad comments that I would influence other girls with immoral activities*.” (17 years grade 9 pupil).

### Lack of family support

Some girls revealed that they had to fulfill multiple roles as learners, mothers and wives. Adolescent school going mothers reported that their family members had to decide that the girls be married. The girls who got married mentioned that it was difficult for them to concentrate on school work as they had to do other house chores as well as taking care of the baby.

*… “I feel victimized by my husband because sometimes you find that I want to start off for school then he would want to sleep with me [having sex]. I have lost weight because I have to do all the house chores, taking care of the baby and studying…I get so tired that most of the times I doze in class. I am not at peace. Sometimes even if I tell him that I am sick, he would want to have sex”.* (19 years grade 12 married pupil).

Furthermore, most adolescent mothers who have been forced into marriage mentioned that their parents/guardian do not have a stable income. Some girls reported that their parents preferred to marry them off rather than keeping both the mother and the child at home.

*“My family have been insisting that I get married. The family to the baby’s father did not want me to get married into their family. It has really been a challenge because some are forcing me to get married; the other side was telling me that I can’t get married to their son…that is how I even started selling homemade cobra so that I pay for my school because I didn’t want to stop school because of my husband”.* (18 years grade 11 pupil).

### Staying away from home

Some girls mentioned having been supported by their parents and being taken care of during pregnancy and after giving birth. However, most adolescent school going mothers’ guardians had no kind words for young mothers, so the girls preferred staying away from home because they were forced to find the person responsible for the pregnancy. They did not want to start a new life with the father of their child. Young mothers mentioned that they felt neglected by the family who did not show any sympathy at all.

*“From the time I got pregnant my parents have had no kind words with me such that when I knock off from school I would rather be with my friends for a while than going home…sometimes I never used to go home from school and they would not care…so my elder sister would take care of the baby. It was difficult to stay at home because of the way they treat me, I felt bad … My mother would always force me to call the father of my child and ask him to bring money. But each time I called him, he would ask for sex, so I was scared to tell my mother” (*17 years grade 9 pupil).

## Consequences of the adolescent mothers’ experiences

### Resilience

The girls reported that the only way they could complete their education was to ignore any comments. Few girls mentioned that they proudly announced having a child to their friends in class. Asked why they were announcing, one girl reported feeling bad the way her classmates were talking about them, so by doing that, she mentioned that it was one way of challenging them that she was not ashamed of what she did but being pained by it.

*“I tried not to pay attention to what others were saying because if I paid attention I would have stopped school. I know that in the end, I am going to live a better life with my child through education. I pretended as if there was nothing wrong and concentrate on my books because if I had concentrated on what they were saying I would have stopped school.”* (18 years grade 11 pupil).

### Skipping some days at school

All the girls mentioned that they had to be absent from school for at least 1 day every month to attend to the child. Some of the girls revealed having missed some days at school just to avoid embarrassment especially immediately after giving birth. Some girls mentioned having experiences of breast milk coming out while in class and her friends would be laughing at her.

*“I would stay away from school just to avoid hearing bad comments from the teachers and my classmates especially the experience of having breast milk coming out...I would dodge at break time …”* (17 years grade 9 pupil).

### Beer drinking

Adolescent school going mothers revealed that they felt embarrassed to associate with other friends as they had nothing in common to talk about. The girls revealed that they demonstrated the behavior of beer drinking due to denial, self-pity and low self-esteem. When asked where they found the money to buy beer, one of the girls who mentioned beer drinking disclosed.

*“I really didn’t know what to do the time I discovered I was pregnant…all I wanted that time was chibuku* [local bulky beer]*. It has helped me up to now to remove stress, people can talk…this time I don’t care whatever they say. It (alcohol) has helped me…Without this, maybe I would have had drunk poison*.” (18 years grade 10 pupil).

### Isolation and rejection

The girls were prone to being lonely and isolated, mostly because of the negative feedback from fellow pupils. Most of the girls were shunned by their friends for fear of being classified in the same category as adolescent school going mothers. The rapport between adolescent school going mothers and their classmates/peers was normally poor.

*“All my friends that I used to play with before I became pregnant had distanced themselves from me….I tried to follow them one time during break, but the way they behaved towards me when I joined them was not good because they all laughed and left me alone…that is how I stopped following them.”* **(**17 years old grade 10 pupil).

## Discussion

In this study, we aimed at exploring the experiences of adolescent school going mothers when they return to school. We have found that adolescent school going mothers have challenges. Teachers demanded that adolescent school going mothers pay the teacher if they were to be helped with the missed lessons. The poor learning environment was found to be one of the challenges adolescent school going mothers faced. This was because adolescent school going mothers were misunderstood by non-parenting girls. Adolescent school going mother were also forced into early marriages by their parents/guardians. The community further perceived schools to have lost values for allowing adolescent mothers back into school. This was seen as encouraging immorality in schools. Non-parenting girls in school did not want to associate with adolescent school going mothers as they were considered to be naughty. We also found that adolescent school going mothers involved themselves in elicit behavior such as beer drinking.

This data were collected from adolescent school going mothers only. Therefore, the results could have been biased because of some strengths and limitations. To begin with, data were collected from two schools in an urban area [Lusaka province only] as the study setting and limited to an urban environment. The study population was a limitation as data were collected from adolescent school going mothers only, leaving out teachers, non-parenting pupils and the community. Generalizability of the findings of the study is a limitation in most studies which is an exception to this study. However, in-depth interview as a qualitative method of gathering information was ideal as respondents were able to express themselves and probing questions were asked after respondents gave their initial response. Furthermore, a phenomenological study was used as it would be better if a mixed method was conducted in this study.

We found that the teachers’ negative attitude made it difficult for adolescent school going mothers to catch up on lessons missed the times they would be absent from school to attend to the baby as teachers demanded that they teach the mothers at a fee. According to Chigona and Chetty [9] teachers were not willing to go through the lesson when a learner has missed due to infant related problem. This has been outlined in other studies conducted in the same region that had the same study population in their studies [10, 17, 18]. Olivier [19] further disputed that some teachers considered adolescent mothers’ issue as private that does not concern them. We further observed that adolescent school going mothers are absent from school at least every month as also observed elsewhere [9] albeit teachers having no flexible timetable for them. Our study suggests the need for the policymaker to introduce workshops for teachers on how best to assist adolescent school going mothers and involve other organizations like health sectors and social welfare to obtain help.

Even if adolescent school going mothers continued with their education, our finding reported that the learning environment for adolescent school going mothers was not good. This is because schools have no committees which are responsible to protect adolescent school going mothers’ welfare especially in cases where they experience any form of humiliation within the school premises. This coincides with earlier reports by Ministry of Education in South Africa [20] that reported that adolescent mothers complained that their schools had not allowed them to attend classes because they were mothers. This is because schools feared that allowing adolescent mothers in school would seem like encouraging immorality in school which is like degrading the education system. This confirms with Chigona and Chetty [9] who revealed that teenage mothers contaminate other non-parenting learners leading to the rejection of those teenage mothers who exercise their education rights by returning to school. Chauke [21] advocated that adolescent school going mothers suffered from anxiety and isolation, as girls in classes did not want to share a seat with adolescent mothers for fear of adulteration [22]. The study suggests practical intervention to build communication between adolescent school going mothers and the school authorities through the formation of committees to be responsible for adolescent school going mothers’ challenges. The poor learning environment for adolescent school going mothers may discourage the young mothers from attending lessons regularly.

Consequently, our study adds to this literature by showing that adolescent school going mothers were forced into early marriages by their parents who withdrew from paying school fees for adolescent school going mothers. The reasons for this could be that parents would not want to continue supporting young mothers with school as having a baby while at school is a sign of not wanting school. This confirms with earlier reports by Jewkes et al. [23] who reported that if society perceives adolescent mothers as women, it is a challenge for them to go back to school. Grant et al. [3] advocated that the birth of the baby marks the end of schooling for adolescent mothers [3] who are often married and their birth may be welcomed by family and society, and therefore, does not involve a social stigma [24]. Explanations for exclusionary practices of young mothers responded to these in strategic ways, where the girls would look for small rented houses and resort to street vending to provide for their baby as well as school. In this regard, Makibi et al. [25] further reported that some families distanced themselves from teen mother because they felt ashamed that the community would look down upon the family for the action of their daughter. The reasons for this was outside the scope of this study because parents were not part of the study sample and the study relied on adolescent mothers for all the information. This study advocates a need to train parents to understand the progressing cognitive capacity of adolescent mothers and help young mothers to advise them of the hardship of being in marriage while at school. We also know that the community is not comfortable to allow adolescent school going mothers in school.

Despite parents of adolescent mothers forcing them into marriage, we have further found that the community members regarded the education system to have lost value because of allowing adolescent mothers in school together with non-parenting girls which is perceived as encouraging immorality in schools. The community would indirectly tease young mothers by calling them with all sort of bad names to discourage them from attending school. Although issues concerning community members forcing the school authorities not to allow adolescent mothers in school were not mentioned in this study, we still argue that this is possible because it has been reported somewhere else in the region. Chigona and Chetty [9] reported that committees in the community were up in arms with the school authority demanding the expulsion of young mothers, where the girls were regarded as ‘othered’ [17, 21, 26]. This is confirmed by Chapati, Yahoo Contributor Network [27] when he explained that teenage mothers are tagged as bad news in Nigeria as it is the case in South Africa, and they are socially ostracized. It was also revealed by Chauke [21] that parents warn their daughters to stay away from the mothers as they feared that their kids will be corrupted by the immoral ways of a teenage mother. The study demonstrates that issues concerning adolescent mothers could incorporate community-based interventions that transmute social–ethnic values associated with how society perceives adolescent school going mother and sexuality. This is observed in an approach that considers the wider social environment of adolescents and acknowledges that the experience of adolescents is influenced by factors located at the individual, family, peer, community and structural levels in determining relevant intervention [28].

While it is possible that adolescent school going mothers may endure with their situation by seeking God’s intervention as found in our study, we also observed that the negative psychological outcomes of being a young mother caused the girls to handle their situation differently where young mothers engaged themselves in illicit behaviors such as beer drinking. This coincides with Huang et al. [29], who reported that most of the teen mothers resort to beer drinking, drug abuse as well as prostitution. The reasons for this are unclear and were beyond the scope of this study but need further exploration particularly given that adolescent mothers resorted to seeking God’s intervention as they struggled with their new role and school [23]. This is confirmed by Zondo [30] who reported that teenage mothers experienced difficulties in balancing their educational responsibilities and taking care of their babies. Smith battle [31] also advocated that substance use was high among the girls. Policy makers in Zambia have not integrated new programs to guide the schools about the well-being of the young mothers. Our study suggests the need for developing strategies to counsel young mothers and parents so that they better understand the developing cognitive capacity of adolescent mothers to understand the meaning of being a mother and to better know when adolescents are in a state of denial so that they don’t involve themselves in illicit behavior.

## Recommendations

Since the study was conducted in an urban area in one district, a similar study should be conducted in a rural area where teachers, parents and the community could be part of the study population. A study should use a mixed method.

## Conclusion

The research was conducted to provide information on experiences of adolescent school going mothers. Our findings show that inadequate support from the teachers, community and fellow pupils disempower adolescent mothers. Adolescent school going mothers are forced into early marriages and therefore involved themselves in street vending while others worked as maids. The community looked down on adolescent mothers calling them all sorts of bad names. It also led young school going mothers to develop certain behaviors such as beer drinking, skipping school days in order to withstand the situation and continue with their education. There is need to strengthen the education system by supporting adolescent mothers through providing flexible time tables where extra lessons could be conducted for free. There is also need to educate the teachers, non-parenting girls and the community on how best to handle adolescent school going mothers and support them through counselling. There is need for policy makers to introduce separate classes and committees to deal with adolescent school going mothers’ problems in order to create a friendly learning environment for adolescent mothers.

## Abbreviations

IDI: In-depth Interviews
VCT: Voluntary Counselling and Testing
